# Efficacy and Safety of Ixazomib Plus Lenalidomide and Dexamethasone Following Injectable PI-Based Therapy in Relapsed/Refractory Multiple Myeloma

**DOI:** 10.1007/s00277-023-05212-7

**Published:** 2023-06-21

**Authors:** Yu Abe, Makoto Sasaki, Naoki Takezako, Shigeki Ito, Kazuhito Suzuki, Hiroshi Handa, Takaaki Chou, Takahiro Yoshida, Ikuo Mori, Tomohiro Shinozaki, Kenshi Suzuki

**Affiliations:** 1 Division of Haematology, Japanese Red Cross Medical Centre, 4 Chome-1-22 Hiroo, Shibuya City, Tokyo, 150-8935 Japan; 2 Division of Haematology, Department of Internal Medicine, Juntendo University School of Medicine, Tokyo, Japan; 3 Division of Haematology, Japan Association for Development of Community Medicine, Nerima Hikarigaoka Hospital, Tokyo, Japan; 4 Division of Haematology and Oncology, Department of Internal Medicine, Iwate Medical University School of Medicine, Iwate, Japan; 5 Division of Clinical Oncology and Haematology, Department of Internal Medicine, The Jikei University Kashiwa Hospital, Chiba, Japan; 6 Department of Haematology, Gunma University Graduate School of Medicine, Gunma, Japan; 7 Niigata Kenshin Plaza, General Incorporated Foundation, Health Medicine Prevention Association, Niigata, Japan; 8 Japan Medical Affairs, Japan Oncology Business Unit, Takeda Pharmaceutical Company Limited, Tokyo, Japan; 9 Department of Information and Computer Technology, Faculty of Engineering, Tokyo University of Science, Tokyo, Japan

**Keywords:** ixazomib, multiple myeloma, proteasome inhibitor, relapsed/refractory disease

## Abstract

**Abstract:**

This nationwide, multicenter, open-label, single-arm study evaluated the efficacy and safety of the oral proteasome inhibitor (PI), ixazomib plus lenalidomide (LEN) and dexamethasone (DEX) (IRd) following injectable PI-based therapy for relapsed/refractory multiple myeloma (RRMM). Of 45 patients enrolled, 36 patients received IRd after achieving at least a minor response to 3 cycles of bortezomib or carfilzomib plus LEN + DEX (VRd, n=6; KRd, n=30). At median follow-up of 20.8 months, the 12-month event-free survival rate (primary endpoint) was 49% (90% CI: 35.9−62.0), counting 11 events of progressive disease/death, 8 dropouts and 4 missing response data. The 12-month progression-free survival (PFS) rate by Kaplan-Meier analysis (dropouts as censoring) was 74% (95% CI: 56−86). Median PFS and time to next treatment (95% CI) were 29.0 (21.3−NE) and 32.3 (14.9−35.4) months, respectively; median OS was not evaluable. The overall response rate was 73%, and 42% of patients had a very good partial response or better. Frequent (≥10% incidence) grade ≥3 treatment emergent adverse events were decreased neutrophil and platelet counts (n=7 [16%] each). Two deaths occurred (one during KRd treatment and one during IRd treatment), both due to pneumonia. IRd following injectable PI-based therapy was tolerable and efficacious in RRMM patients.

**Trial registration number:**

NCT03416374; Date of registration: January 31, 2018

**Supplementary Information:**

The online version contains supplementary material available at 10.1007/s00277-023-05212-7.

## Introduction

The introduction of proteasome inhibitors (PI) has drastically improved the prognosis for transplant-eligible and -ineligible patients with newly diagnosed (NDMM) and relapsed/refractory MM (RRMM) [1–4]. PI-based therapy has been shown to improve both progression-free survival (PFS) and overall survival (OS) compared with non-PI-based therapy in global phase 3 clinical trials [1–3], and several PIs have now been approved and are widely used in routine clinical practice in MM. Recently, there has been a shift in the treatment paradigm towards the use of extended, continuous PI-based therapy, which has delivered superior outcomes compared with shorter, fixed-duration therapy [5].

PI-based triplet regimens comprising the injectable PI, bortezomib (VRd) or carfilzomib (KRd), or the oral PI, ixazomib (IRd), with a lenalidomide and dexamethasone (Rd) backbone are approved and routinely used for the treatment of RRMM in Japan. However, physical, social, and geographic barriers to health service access, burden of repeated administration of injectable drugs, and toxicity may interfere with the long-term delivery of PIs in clinical practice. Both bortezomib and carfilzomib are associated with the development of peripheral neuropathy [6] and cardiotoxicity [7], which are related to cumulative dose. Frequent outpatient visits and hospitalizations may be required during periods of high MM disease activity to control symptoms and manage AEs following initiation of treatment, but once the disease has stabilized, oral treatment options should also be considered [8]. This view is supported by the findings from several studies, which have shown that cancer patients prefer oral over intravenous (IV) administration for reasons including convenience, perceived efficacy, and past experience [8].

The oral PI, ixazomib, is now approved in over 60 countries [9]. In Japan, ixazomib was approved in 2017 [10] in combination with Rd (IRd) for patients with RRMM, and in 2021 as monotherapy for patients with MM as maintenance therapy. A US-based, phase 4, real-world MM-6 study is currently being conducted in patients with NDMM who were previously treated with bortezomib-based therapy prior to enrolment [11]. The purpose of both the MM-6 study and our study is to evaluate the efficacy and safety of switching to oral PI-based treatment, including potential benefits in both patient and health resource burden. It is hoped that switching to IRd will facilitate continuous PI treatment, whilst ensuring clinical outcomes achieved with injectable PI-based treatment are maintained. Although an interim analysis of the ongoing MM-6 study has recently reported the efficacy and safety of switching to IRd in patients with NDMM; to date, the efficacy and safety of switching to IRd following injectable PI-based therapy has not been evaluated in the RRMM setting.

Here, we report the results from a nationwide, multicenter, open-label, single-arm study evaluating the efficacy and safety of treatment with IRd therapy after 3 cycles of injectable PI-based, VRd or KRd, therapy in patients with RRMM from 17 study sites in Japan.

## Methods

### Study Design and Patients

This was a nationwide, multicenter, open-label, single-arm study conducted in Japanese patients with RRMM in Japan (NCT03416374). Detailed eligibility criteria are provided in Online Resource 1. Briefly, eligible patients were adults aged ≥20 years with RRMM who were not refractory to bortezomib, carfilzomib, or lenalidomide and were considered transplant-ineligible by the investigator, or if considered transplant-eligible, were not planning to undergo stem cell transplantation for ≥12 months. Also, patients must have been planning to start combination therapy with VRd or KRd as second-, third-, or fourth-line therapy, and have an Eastern Cooperative Oncology Group Performance Status (ECOG PS) score of 0−2 (or an ECOG PS of 3 if symptoms were associated with bone lesions only).

Eligible patients received VRd or KRd therapy for 3 cycles. A standard dose of each drug was recommended as per the package insert; however, dosage adjustments were permitted for select patients at the investigator’s discretion. Patients who achieved at least a minor response (MR) to VRd or KRd transitioned to IRd therapy. After transition, oral administration of IRd therapy at a dosage of 4 mg ixazomib on days 1, 8, and 15 plus 25 mg lenalidomide on days 1 through 21, and 40 mg dexamethasone on days 1, 8, 15, and 22, was recommended in 28-day cycles. Dosage adjustments for lenalidomide were permitted in patients with renal impairment, as per the package insert [12]. Dose interruptions/modifications of all three drugs were permitted for toxicities suspected to be related to the specific drugs.

Study drug administration was discontinued if the patient did not meet criteria for transition to IRd treatment (Online Resource 1), or in the event of withdrawal of consent, progressive disease (PD), unacceptable toxicity, significant protocol deviation, death, dropout or loss to follow-up, or if the investigator deemed it necessary. IRd was continued until PD or unacceptable toxicity.

The trial was designed by the steering committee members and the secondary sponsor (Takeda). The protocol was approved by the Certified Review Board and registered with the Ministry of Health, Labour and Welfare (Japan Registry of Clinical Trials). All patients provided written informed consent to participate in the study.

## Endpoints

The primary endpoint was the 12-month event-free survival (EFS) rate from the first dose of injectable PI-based therapy, with dropouts, missing response data, PD, and deaths considered as events. Secondary endpoints included: PFS (defined as the period between the first dose of injectable PI-based therapy until the date of confirmed PD or death due to any cause, whichever occurred first) by Kaplan-Meier analysis, with dropouts treated as censoring; OS (defined as the period between the first dose of injectable PI-based therapy until the date of death due to any cause); rate of minimal residual disease (MRD) negativity in bone marrow (BM) in patients who achieved complete response (CR), with MRD negativity defined as the absence of tumour plasma cell within 100 000 BM cells (i.e. <10^-5^) by single-tube 8-colour multiparameter flow cytometry (MFC) method (SRL-Flow) [13] or 1 000 000 BM cells (i.e. <10^-6^) by adaptive next-generation sequencing (NGS) [14]; best response (CR, very good partial response [VGPR], partial response [PR], MR, stable disease, PD); overall response rate (ORR; the proportion of patients achieving ≥PR); the proportion of patients achieving VGPR or better; duration of response (DOR; defined as the period between the date of first PR or better and the earliest date of PD or death due to any cause); time to next treatment (TTNT), defined as the period between the first dose of injectable PI-based therapy to the date of the next antitumor treatment or death due to any cause, whichever occurred first; health-related quality of life (HRQOL) and Quality Adjusted Life Years (QALY); healthcare resource utilization (HCRU), including the length of hospital stay and outpatient visits (per person-month) during the initial 3 cycles of injectable PI-based therapy and following IRd treatment; relative dose intensity (RDI) for each IRd study drug (see Online Resource 2 for full description); and safety.

## Assessments

Responses were assessed according to the International Myeloma Working Group Uniform Response Criteria (2016) [14]. M-protein in serum and urine was measured at scheduled study visits, with substitution to serum free light chain permitted. BM aspiration was performed in patients suspected of achieving CR during IRd treatment for evaluation of the presence of MRD. HRQOL was assessed using the European Organisation for Research and Treatment of Cancer (EORTC) Quality of Life Questionnaire (QLQ)-C30 (v3.0) and EORTC QLQ-MY-20 questionnaires. Questionnaires were administered at baseline and every 3 cycles during IRd treatment, and at the end of treatment/discontinuation.

Safety was evaluated by assessment of treatment-emergent adverse events (TEAEs), including Grade ≥3 and serious adverse events (SAEs) during both VRd/KRd and IRd treatment periods. TEAEs were evaluated and categorised by system organ class (SOC) and preferred term (PT) using MedDRA/J version 24.0. Severity of TEAEs were graded according to the Common Terminology Criteria for Adverse Events (CTCAE) version 4.03.

## Statistical Analysis

The full analysis set (FAS) and safety analysis set (SAS) was defined as all patients who were enrolled and received ≥1 dose of VRd or KRd therapy, and were used for efficacy and safety analyses, respectively. Safety was further evaluated using the secondary safety analysis set (SSAS), defined as all patients who received ≥1 dose of IRd treatment. A 12-month EFS rate, the primary endpoint, of 36% was set as the efficacy threshold for hypothesis testing based on the phase 2 study of VRd in patients with RRMM, in which the median PFS was 9.5 months and the 12-month PFS rate by Kaplan-Meier analysis was 36% [15]. A sample size of 39 patients was set to reject the null hypothesis with at least 80% power with a one-sided significance level of 0.05 under the assumed 57% PFS rate, based on the results of the phase 3 study of IRd in RRMM [16]. Factoring in a discontinuation rate of <20% before transition to IRd treatment (which was counted as events in the primary endpoint), the target number of patients was set as 47 to include 39 patients initiating IRd.

The 12-month EFS rate was compared with 36% using the exact binomial test with one-sided significance level of 5% and the corresponding 90% confidence interval (CI) was estimated. Time-to-event analyses of secondary endpoints (PFS, OS, TTNT, and DOR) were performed using the Kaplan-Meier method, with their 95% CIs.

The modified QALY was calculated by converting the global health/QOL scale score from the EORTC-QLQ-C30 during treatment into a utility value ranging from 0 to 1 and multiplying it by the duration of the treatment effect. Baseline demographics and other secondary endpoints (safety data, best response, HCRU, QOL variables) were summarized using descriptive statistics. The mean, standard deviation (SD), and median (min, max) were calculated for continuous variables, and the frequency number and proportion were calculated for categorical variables.

All statistical analyses were performed using SAS® software version 9.4 (SAS Institute, Cary, NC, USA).

## Results

**Fig. 1:**
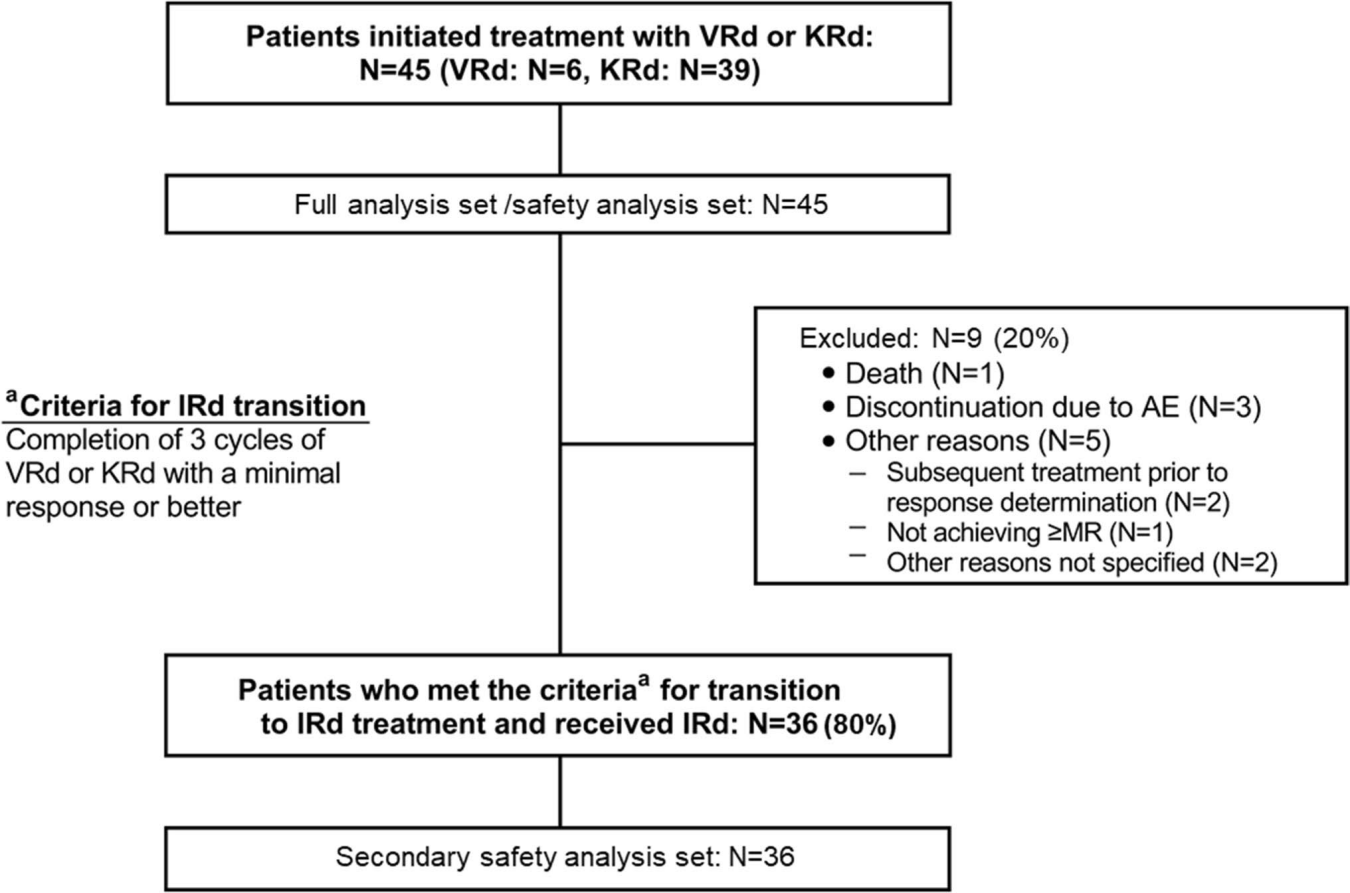
Patient Disposition. AE, adverse event; IRd, ixazomib, lenalidomide, dexamethasone; KRd, carfilzomib, lenalidomide, dexamethasone; VRd, bortezomib, lenalidomide, dexamethasone

Between Feb 28, 2018 and May 28, 2021, 45 patients were enrolled and received ≥1 dose of VRd or KRd and comprised the FAS and SAS, respectively. Of the 45 patients enrolled, 9 patients discontinued during VRd or KRd therapy because of AEs (n=3), death (n=1), and other reasons (n=5) (Fig. 1). Thirty-six patients (VRd, n=6; KRd, n=30) were eligible to transition to IRd treatment and received ≥1 dose of IRd.

Patient demographics and baseline characteristics are presented in Table 1. Of the 45 patients in the FAS, the mean (SD) age was 70.7 (9.2) years, the median (min, max) number of prior therapies was 2 (1, 4), and 73% and 22% of patients had an ECOG PS of 0 and 1, respectively (Table 1). The numbers of patients previously exposed to bortezomib, carfilzomib, lenalidomide and pomalidomide prior to this study were 32 (71%), 4 (9%), 32 (71%), and 6 (13%), respectively. Four (9%) and 2 (4%) patients had received prior antibody therapy with elotuzumab and daratumumab, respectively.

**Table 1:** Patient Demographics and Baseline Characteristics (Full Analysis Set)

	FAS *N*=45
Age, years, median (min, max)	70 (37, 85)
≤65	10 (22)
>65−≤75	21 (47)
>75	14 (31)
Sex, female	21 (47)
ISS stage at initial diagnosis	
I/ II/ III	19 (42)/ 17 (38)/ 9 (20)
ECOG PS	
0/ 1/ 2	33 (73)/ 10 (22)/ 2 (4)
Presence of peripheral neuropathy	
Grade 1	7 (16)
M-protein subtype at initial diagnosis	
IgG	26 (58)
IgA	5 (11)
Bence Jones	10 (22)
Other	4 (9)
Prior lines of therapy, median (min, max)	2.0 (1.0, 4.0)
1/ 2/ 3/ 4	22 (49)/ 14 (31)/ 8 (18)/ 1 (2)
Prior therapies	
Radiation therapy	4 (9)
HSCT	19 (42)
Prior PI therapy	
Bortezomib	32 (71)
Carfilzomib	4 (9)
Prior IMiDs therapy	
Lenalidomide	32 (71)
Pomalidomide	6 (13)
Prior antibody therapy	
Daratumumab	2 (4)
Elotuzumab	4 (9)
Cytogenetic abnormalities at initial diagnosis*	
t (4;14)	5 (11)
t (14;16)	0
del17p	6 (13)

Data presented as n (%) unless otherwise specified. Percentages may not add up to 100 due to rounding

ECOG PS, Eastern Cooperative Oncology Group Performance Status; HSCT, haematopoietic stem cell transplantation; ISS, International Staging System; KRd; carfilzomib, lenalidomide, and dexamethasone; N/A, not available; PI, proteasome inhibitor; VRd, bortezomib, lenalidomide, and dexamethasone, IMiDs, immunomodulatory drugs

*All cytogenetic abnormalities were based on voluntary reporting of evaluation results at each study site according to the standard methods and positive cut-offs of each site

The median duration of follow-up was 20.8 (95% CI: 17.4−23.7) months.

## Efficacy

### Primary Endpoint

The 12-month EFS rate was 49% (90% CI: 35.9−62.0) (22/45 patients), which exceeded the threshold of 36%, but the p value (p=0.0518) did not reach the pre-determined level of significance (p=0.05). The counted events were 9 events of PD, 2 deaths, 8 dropouts (discontinuation due to AEs, n=5; not achieving ≥MR during injectable PI treatment, n=1; and other reasons n=2), and 4 patients with missing response data.

### Secondary Endpoints

**Fig. 2:**
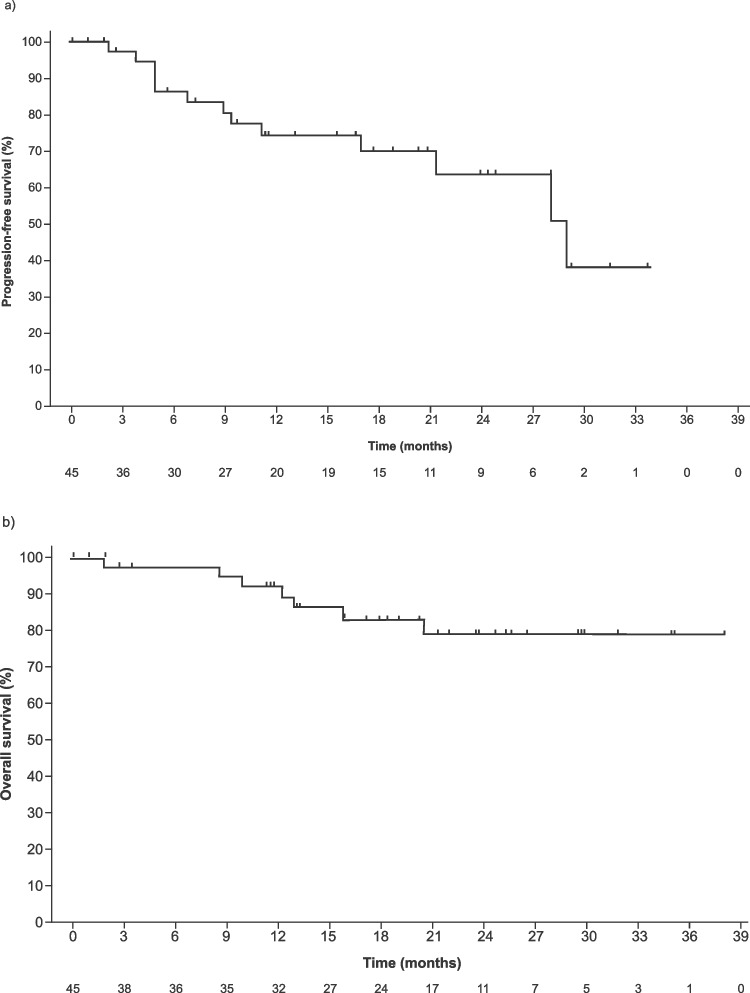
Progression-Free Survival (**a**) and Overall Survival (**b**) During Study Treatment. IRd, ixazomib, lenalidomide, dexamethasone; KRd, carfilzomib, lenalidomide, dexamethasone; VRd, bortezomib, lenalidomide, dexamethasone

**Fig. 3:**
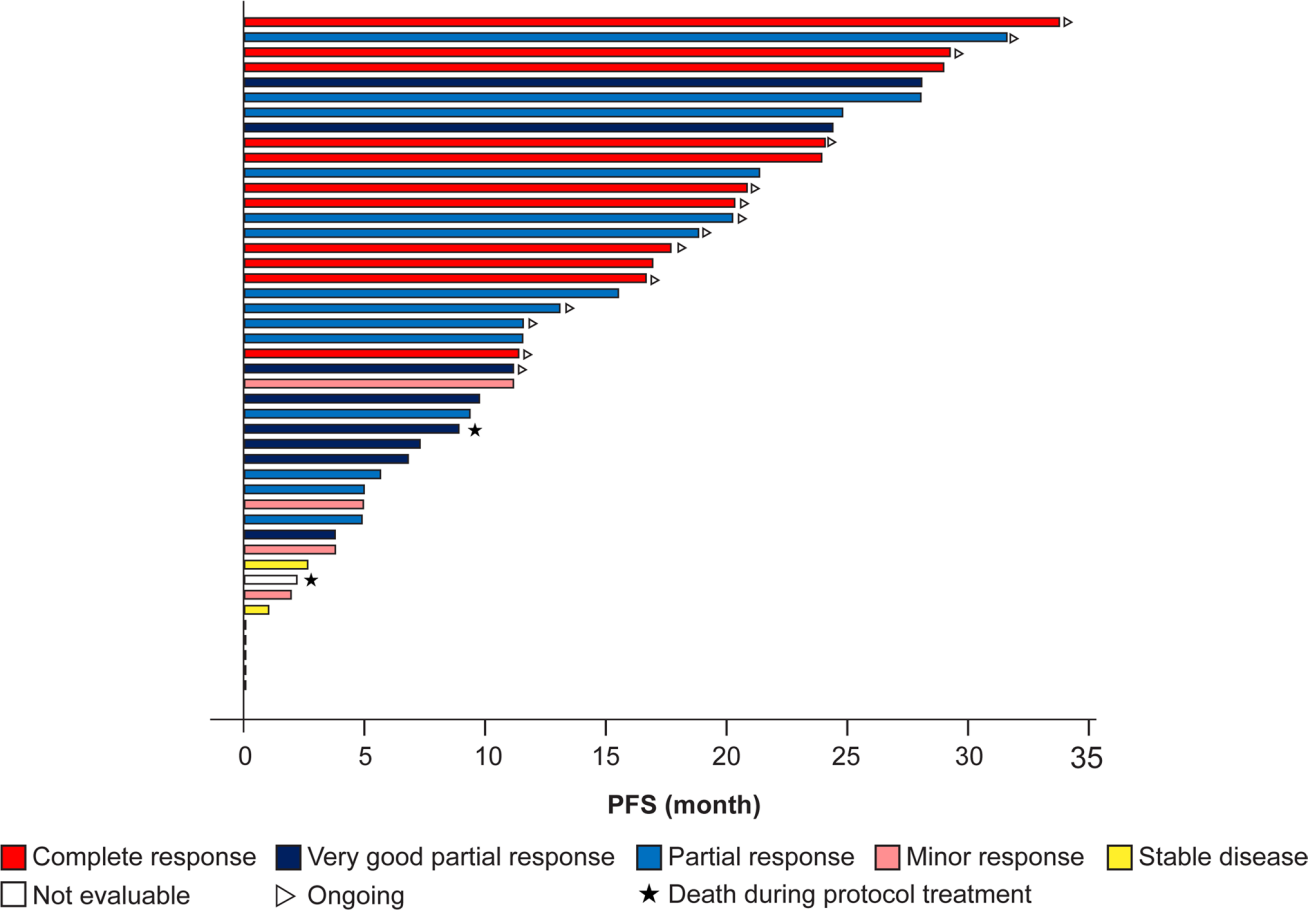
Swimmer Plot of Progression-Free Survival During Study Treatment

**Fig. 4:**
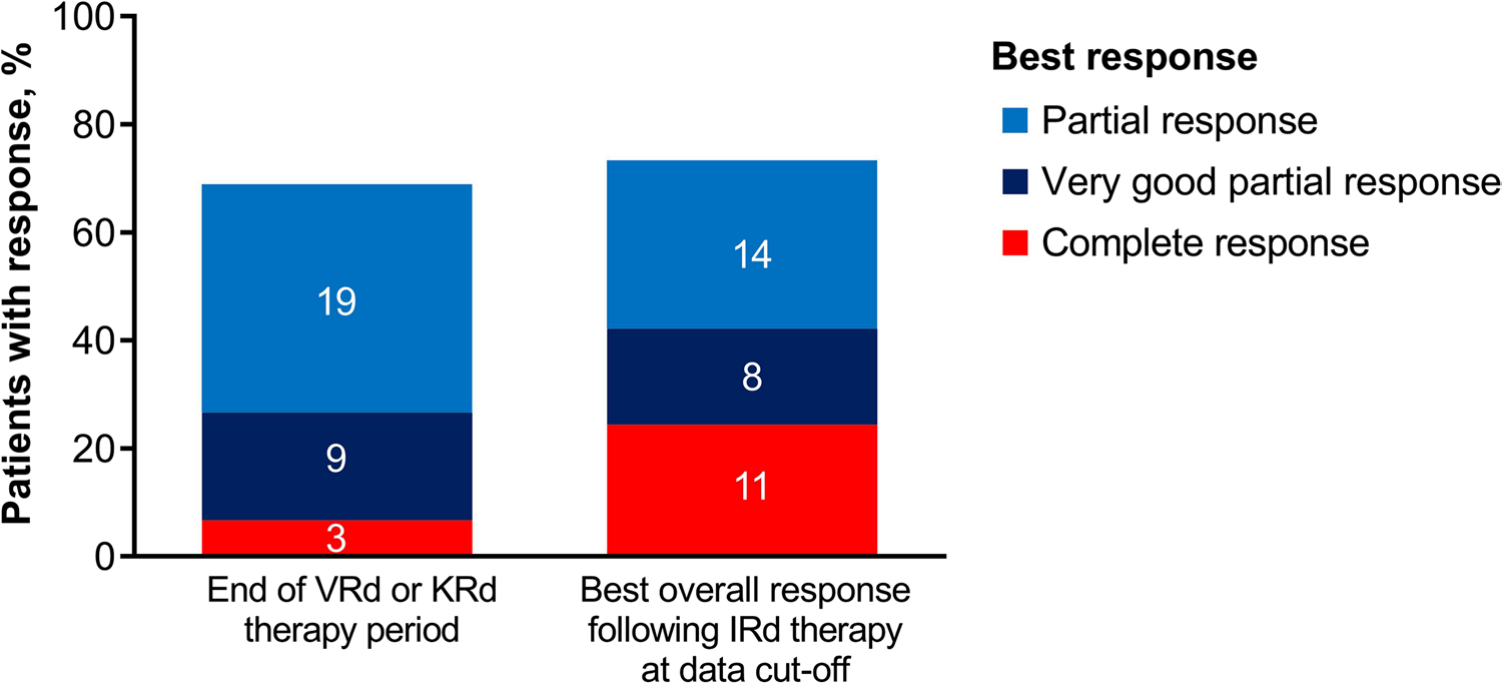
Best Overall Response Rate with Injectable-PI Based Therapy and Following IRd Therapy. IRd, ixazomib, lenalidomide, dexamethasone; PI, proteasome inhibitor. Number of patients with a response are presented within each column

The 12-month PFS rate by Kaplan-Meier analysis (dropouts as censoring) was 74% (95% CI: 56−86) and the median PFS was 29.0 (95% CI: 21.3−NE) months; the median OS was not estimable. The median TTNT and DOR were 32.3 (95% CI: 14.9−35.4) months and 28.0 (95% CI: 20.4−NE) months, respectively. Figure 2 presents PFS and OS in the FAS. A swimmer plot summarizing treatment outcomes for all 45 patients is shown in Fig. 3. The ORR was 73% (95% CI: 58.1−85.4) and the rate of VGPR or better was 42% (95% CI: 27.7−57.8). The best overall response to study treatment was CR in 11 (24%) patients, VGPR in 8 (18%) patients, PR in 14 (31%) patients, MR in 4 (9%) patients, stable disease in 2 (4%) patients, and not evaluable in 6 (13%). Further, the number of patients achieving CR or VGPR increased during IRd treatment (Fig. 4; Online Resource 3).

Of the 11 patients who achieved CR, 7 patients were MRD negative by the SRL-flow method (<10^-5^) and 3 patients were MRD negative by the NGS method (<10^-6^). MRD was not measurable by the NGS method in 2 patients. The length of hospital stay, and outpatient visits during the initial 3 cycles of injectable PI-based treatment and following IRd treatment were 5.3 vs 1.0 days per person-month, and 4.2 vs 1.9 visits per person-month, respectively.

The median duration of treatment was 12.4 (95% CI: 8.86−24.79) months. The mean (SD) RDI of ixazomib, lenalidomide, and dexamethasone treatment during IRd therapy was 81% (16.4), 53% (30.5), and 43% (31.8), respectively (Online Resource 4). HRQOL was maintained for the duration of the study treatment (Online Resource 5). With the exception of diarrhea, disease symptoms scores were also maintained. The QALY was 0.518 at 12 months after the start of the study.

## Safety

Safety was analysed in 45 patients in the SAS and 36 patients in the SSAS. An overview of the safety profile during IRd therapy (SSAS) and overall from the start of injectable PI-based therapy (SAS) is presented in Table 2. The incidence of common (with an incidence of ≥5%) all grade TEAEs and grade ≥3 TEAEs are presented in Table 3. TEAEs occurred in 41 of 45 patients (91%) in the SAS and in 28 of 36 patients (78.0%) in the SSAS. The most common (≥5 patients) TEAEs of any grade in the SSAS were diarrhea (n=11 [31%]), white blood cell count decreased (n=9 [25%]), and pneumonia (n=5 [14%]). Grade ≥3 TEAEs occurred in 15 of 36 patients (42%) in the SSAS, including white blood cell count decreased, pneumonia and platelet count decreased which were the most common (n=4 [11%] each).

**Table 2:** Overview of Safety Profile During IRd Therapy (Secondary Safety Analysis Set) and Injectable PI-Based Therapy for 3 cycles Plus IRd Therapy (Safety Analysis Set)

TEAE	SAS *N*=45	SSAS *N*=36
	Patients, *n* (%)	Patients, *n* (%)
Any grade TEAE	41 (91)	28 (78)
Any grade IRd-related TEAE	N/A	21 (58)
Grade ≥3 TEAE	25 (56)	15 (42)
Grade ≥3 IRd -related TEAE	N/A	10 (28)
TEAE leading to discontinuation of IRd therapy	N/A	6 (17)
Serious TEAE	17 (38)	11 (31)
Deaths during study	2 (4)	1 (3)

SAS, safety analysis set; SSAS, secondary safety analysis set; TEAE, treatment-emergent adverse event.

**Table 3:** Occurrence of Common (with a PT incidence of ≥5%) TEAEs During IRd Therapy (Secondary Safety Analysis Set) and Injectable PI-Based Therapy for 3 cycles Plus IRd Therapy (Safety Analysis Set)

TEAE (PT incidence of ≥5%)	SAS *N*=45		SSAS *N*=36	
	Grade, *n* (%)		Grade, *n* (%)	
	Any grade	Grade ≥3	Any grade	Grade ≥3
Diarrhea	13 (29)	0	11 (31)	0
White blood cell count decreased	11 (24)	4 (9)	9 (25)	4 (11)
Pneumonia	5 (11)	4 (9)	5 (14)	4 (11)
Platelet count decreased	9 (20)	7 (16)	4 (11)	4 (11)
Neutrophil count decreased	7 (16)	7 (16)	4 (11)	3 (8)
Constipation	4 (9)	0	3 (8)	0
Malaise	4 (9)	0	3 (8)	0
Nasopharyngitis	4 (9)	0	3 (8)	0
Decreased appetite	3 (7)	0	3 (8)	0
Taste disorder	3 (7)	0	3 (8)	0
Thrombocytopenia	2 (4)	2 (4)	2 (6)	2 (6)
Lymphocyte count decreased	2 (4)	2 (4)	2 (6)	2 (6)
Cataract	2 (4)	1 (2)	2 (6)	1 (3)
Nausea	2 (4)	0	2 (6)	0
Herpes zoster	2 (4)	0	2 (6)	0
Alanine aminotransferase increased	2 (4)	0	2 (6)	0
Muscle spasms	2 (4)	0	2 (6)	0
Peripheral sensory neuropathy	2 (4)	0	2 (6)	0
Rash	8 (18)	2 (4)	2 (6)	0
Anemia	4 (9)	2 (4)	1 (3)	1 (3)
Pyrexia	5 (11)	1 (2)	1 (3)	0

PT, preferred term; SAS, safety analysis set; SSAS, secondary safety analysis set; TEAE, treatment-emergent adverse event.

Serious TEAEs occurred in 17 of 45 patients (38%) in the SAS. Of these, events that occurred in ≥2 patient were pneumonia (n=5 [11%]), acute kidney injury and influenza (n=2 [4%] each). Two patients died from TEAEs in the SAS. One died due to pneumonia during IRd treatment and it was considered related to study treatment. The other died of bacterial pneumonia, which was not considered to be related to study treatment. During IRd therapy, 6 of 36 patients (17%) developed TEAEs that led to treatment discontinuation. Five patients discontinued due to diarrhea, gastroenteritis, pneumonia, tibia fracture and interstitial lung disease, respectively. One patient discontinued because of both decreased appetite and taste disorder. Peripheral neuropathy occurred in 3 (8%) patients during IRd treatment. Two (6%) of which were considered related to study treatment; both of these events were grade ≤2.

## Discussion

This nationwide, multicenter, open-label, single-arm study was designed to control patients' disease early in treatment with three cycles of injected PI, reduce side effects such as peripheral neuropathy and cardiotoxicity, and reduce the burden of long-term parenteral therapy. Using this approach, the favourable efficacy of IRd therapy following VRd or KRd therapy in patients with RRMM was demonstrated in the real-world setting. The median PFS was 29.0 months, the estimated 12 month PFS rate was 74% when analysed by the Kaplan-Meier method regarding dropouts as censoring, and the ORR was 73%.

The PFS benefit and ORR observed in our study were comparable with those reported with PI-based therapies in other clinical trials in RRMM (Table 4), including the phase 3 study of KRd (median PFS, 26.3 months; ORR: 87.1%),[17] the phase 3 study of IRd (median PFS, 20.6 months; ORR: 78%) [16], and the phase 2 study of IRd (median PFS, 22.0 months; ORR: 84.4%) in Japanese patients [18], despite differences in patient demographics and other background factors. For example, the median age (70.0 years) and proportion of elderly patients (>65 years: 77.8%) in our study was higher than in the phase 3 KRd study [17] (median age: 64.0 years; ≥65 years: 46.7%), and phase 3 [16] (median age: 66 years; >65yr: 53.3%) and Japanese phase 2 [18] (median age: 67 years; >65 years: not reported) IRd studies. There was also a trend towards a higher number of regimens in our study (1 regimen: 49%; 2 regimens: 31%; 3 regimens: 18%; ≥4 regimens: 2%), compared with the phase 3 KRd study [17] (1 regimen: 46.5%; 2 or 3 regimens: 53.3%) and phase 3 [16] (1 regimen: 62.2%; 2 regimens: 26.9%; 3 regimens: 10.8%) and Japanese phase 2 [18] (1 regimen: 61.8%; 2 regimens: 35.3%; 3 regimens: 2.9%) IRd studies. Nevertheless, the PFS and ORR benefits were comparable.

**Table 4:** Summary of Pivotal Studies of Proteasome Inhibitor-Based Triplet Regimen in RRMM

Study/NCT ID/Phase	Regimen	Key efficacy data	Three most common adverse events among all grade and grade 3-4 toxicities
**IRd vs Rd** TOURMALINE-MM1 NCT01564537 Phase 3 Moreau et al. *NEJM* 2016	28D cycles **I** 4 mg D1,8,15 **R** 25 mg D1–21 **d** 40 mg D1,8,15,22	mPFS 20.6 (IRd) vs 14.7 (Rd) months ORR 78% vs 72% VGPR or better 48% vs 39% mDOR 20.5 vs 15.0 months	All grades, IRd vs Rd: Diarrhea 45% vs 39%, neutropenia 33% vs 31%, constipation 35% vs 26% Grade 3–4: Neutropenia 22% vs 24%, thrombocytopenia 19% vs 9%, anemia 9% vs 13%
**IRd, single arm** NCT02917941 Phase 2 Iida et al. *Int J Clin Oncol* 2022	28D cycles **I** 4 mg D1,8,15 **R** 25 mg D1–21 **d** 40 mg D1,8,15,22	mPFS 22.0 months ORR 84.4% VGPR or better 50.0% mDOR NR	All grades, IRd: Constipation 50%, upper respiratory tract infection 47%, diarrhea 41% Grade 3–4: Neutropenia 21%, Platelet count decreased 21%, Neutrophil count decreased 18%
**VRd, single arm** NCT00378209 Phase 2 Richardson et al. *Blood* 2014	21D cycles **V** 1 mg D1,4,8,11 **R** 15 mg D1–14 **d** 40/20 mg (1–4 cycles); 20/10 mg (5−8 cycles)	mPFS 9.5 months ORR 64% VGPR or better 28% mDOR 8.7 months	All grades, VRd: Sensory neuropathy 53%, fatigue 50%, neutropenia 42% Grade 3–4: Neutropenia 30%, thrombocytopenia 22%, lymphopenia 11%
**KRd vs Rd** NCT01080391 Phase 3 Stewart et al. *NEJM* 2015	28D cycles K* D1,2,8,9,15,16 *starting dose, 20 mg/m^2^ D1,2 of cycle 1; target dose, 27 mg/m^2^ thereafter **R** 25 mg D1–21 **d** 40 mg D1,8,15,22	mPFS 26.3 (KRd) vs 17.6 (Rd) months ORR 87.1% vs 66.7% VGPR or better 69.9% vs 40.4% mDOR 28.6 vs 21.2 months	All grades, KRd vs Rd: Anemia 42.6% vs 39.8%, Diarrhea 42.3% vs 33.7%, Neutropenia 37.8% vs 33.7% Grade 3–4: Neutropenia 29.6% vs 26.5%, Anemia 17.9% vs 17.2%, Thrombocytopenia 16.6% vs 12.3%,

d, dexamethasone; I, ixazomib; K, carfilzomib; mDOR, median duration of response; mPFS, median progression-free survival; NR, not reported; ORR, overall response rate; R, lenalidomide; V, bortezomib; VGPR, very good partial response

On the other hand, based on the conservative analysis method which counted discontinuation due to AEs and missing response data at 12 months as events, the 12-month EFS rate was 49% (22/45) (primary endpoint). The discrepancy between EFS rates observed for the primary and PFS rate for secondary endpoint mainly derived from the 12 events (discontinuation due to AE [n=5], not achieving ≥MR [n=1], other reasons [n=2], and missing response data [n=4]) other than PD and death. Although it had been taken into account in this study’s design, these discontinuations showed the difficulties with management in the early phase of treatment for RRMM. Nevertheless, the 12-month EFS rate exceeded the pre-specified threshold and suggested the effectiveness of IRd treatment after response was achieved and the disease status was stable. That was also supported by the positive findings for the remaining secondary efficacy endpoints, including TTNT (median 32.3 months), DOR (median, 28.0 months), ORR (73%) and proportion of patients with VGPR or better (42%).

A deepening of response was observed with IRd therapy after injectable PI-based induction. Nine (20.0%) patients achieved VGPR or better and 3 (6.7%) patients achieved a CR with VRd or KRd therapy. Following transition to IRd therapy, 19 (42.2%) patients achieved VGPR or better and 11 (24.4%) patients achieved CR. Of these, 7 (15.6%) patients were MRD negative by the SRL-flow method (<10^-5^) and the MRD negativity rate was 19.4% (7/36) by this method, which is considered promising for PI-based rescue chemotherapy that does not target CD38 [19].

The results of our study provide data that can be used as a reference for treatment options for patients with RRMM who have a sufficient response to short-term injectable PIs but desire oral drug management. In global phase 3 clinical trials, PI-based therapy has been shown to improve both PFS and OS compared with non-PI-based therapy [1–3]. However, real-world outcomes with PI-based therapies have rarely matched those achieved under clinical trial conditions in patients with RRMM [13, 20, 21].

The reason for the discrepancy between clinical trial efficacy and real-world effectiveness is most likely due to differences in patient characteristics as a result of the strict eligibility criteria employed for randomized controlled trials (RCTs). This view is supported by data from the US-based Connect-MM registry and other real-world studies in MM, which show that between 22 and 70% of patients with MM would be considered ineligible for participation in RCTs [22–24]. In contrast to RCTs, the patient population seen in clinical practice is typically heterogeneous, with a broader age range, including older and frail patients who are particularly susceptible to toxicities [22].

Importantly, outcomes achieved following transition from injectable PI-based therapies and immunomodulatory drugs under real-world conditions were similar to those achieved in phase 3 clinical studies, including PFS and TTNT (19.2−27.6 vs 17.5−20.6 months, respectively) [20]. Based on the real-world evidence of our study and others [11, 25], transition to ixazomib-based therapy represents an attractive alternative to current regimens for long-term PI-based therapy and may afford patients the similar effectiveness in clinical practice as those seen in RCTs. The length of hospitalization and the number of outpatient visits after changing to oral PI-based therapy were numerically low, which may represent an advantage over injectable PI-based therapy. Treatment strategies such as this study may be useful for patients who may want to reduce their number of hospital visits or for whom hospital visits are an added burden. In addition, QOL was not notably affected throughout the treatment period from injectable PI-based therapy to IRd, and it was confirmed that PI-based treatment was continued. Transition from injectable PI-based therapy to oral PI-based therapy was tolerable in clinical practice, and the safety profile of IRd was similar to, and consistent with previous clinical trial data [16, 18, 26, 27]. TEAEs reported in this study were similar to those reported in the global phase 3 IRd study [16], and no previously unknown safety concerns were identified in this study [28].

This study must also be considered in light of its limitations. Firstly, this was a single-arm study, precluding direct comparisons from being made. The number of patients receiving ixazomib-based therapy in our study was also relatively small, and thus further study in a larger patient population is required to confirm these findings.

In conclusion, this study demonstrated that IRd therapy after injectable PI-based therapy was tolerable and effective for patients with RRMM in the real-world setting. Transition to oral PI-based therapy may be a good option to allow prolonged administration of PI-based therapies, resulting in better outcomes. Our findings add to the evolving body of evidence related to ixazomib and provide additional support for its use.

## Supplementary Information

Supplementary file 1 (DOCX 2.93 mb)

Supplementary file 2 (DOCX 78.7 kb)

Supplementary file 3 (DOCX 220 kb)

Supplementary file 4 (DOCX 3.19 mb)

Supplementary file 5 (DOCX 9.76 mb)

## Statements and Declarations

### Data-sharing statement

The datasets, including the redacted study protocol, redacted statistical analysis plan, and individual participants’ data supporting the results reported in this article, will be made available within three months from initial request, to researchers who provide a methodologically sound proposal. The data will be provided after its de-identification, in compliance with applicable privacy laws, data protection and requirements for consent and anonymization.
