# Correction to: Efficacy and safety of ixazomib plus lenalidomide and dexamethasone following injectable pi‑based therapy in relapsed/refractory multiple myeloma

**DOI:** 10.1007/s00277-023-05355-7

**Published:** 2023-07-11

**Authors:** Yu Abe, Makoto Sasaki, Naoki Takezako, Shigeki Ito, Kazuhito Suzuki, Hiroshi Handa, Takaaki Chou, Takahiro Yoshida, Ikuo Mori, Tomohiro Shinozaki, Kenshi Suzuki

**Affiliations:** 1 Division of Haematology, Japanese Red Cross Medical Centre, 4 Chome-1-22 Hiroo, Shibuya City, Tokyo 150-8935 Japan; 2 Division of Haematology, Department of Internal Medicine, Juntendo University School of Medicine, Tokyo, Japan; 3 Division of Haematology, Japan Association for Development of Community Medicine, Nerima Hikarigaoka Hospital, Tokyo, Japan; 4 Division of Haematology and Oncology, Department of Internal Medicine, Iwate Medical University School of Medicine, Iwate, Japan; 5 Division of Clinical Oncology and Haematology, Department of Internal Medicine, The Jikei University Kashiwa Hospital, Chiba, Japan; 6 Department of Haematology, Gunma University Graduate School of Medicine, Gunma, Japan; 7 Niigata Kenshin Plaza, General Incorporated Foundation, Health Medicine Prevention Association, Niigata, Japan; 8 Japan Medical Affairs, Japan Oncology Business Unit, Takeda Pharmaceutical Company Limited, Tokyo, Japan; 9 Department of Information and Computer Technology, Faculty of Engineering, Tokyo University of Science, Tokyo, Japan

**Correction to: Annals of Hematology**

10.1007/s00277-023-05212-7

The graph of figure 4 needs to be corrected. Although the numbers are correct, the width of the graph on the right is incorrect. The widths of 11 (red) and 14 (blue) on the right graph should be revised.

The original article has been corrected.

