# Supplementary material for: Efficacy and Safety of Ixazomib Plus Lenalidomide and Dexamethasone Following Injectable PI-Based Therapy in Relapsed/Refractory Multiple Myeloma
Source: Ann Hematol. 2023 Jun 21;102(9):2493–504. doi: 10.1007/s00277-023-05212-7 (PMC10444638; doi:10.1007/s00277-023-05212-7)

# Supplementary Table 1. Detailed Eligibility Criteria for the Study Population

| **VRd or KRd Treatment Period** |
| --- |
| **Inclusion criteria** |
| - Males and females aged ≥20 years with RRMM. - Planning to start combination VRd or KRd as second-, third-, or fourth-line therapy. - Measurable disease, defined as ≥1 of:   - Serum M protein ≥0.5 g/dL;   - Urine M protein ≥200 mg/24 hrs;   - Serum free light chain concentration ≥10 mg/dL if ratio is abnormal. - ECOG PS 0−2* (*ECOG PS 3 permitted provided symptoms were associated with bone lesions only). - Transplant-ineligible, or transplant-eligible patients provided that they are not planning to undergo transplant for ≥12 months after the start of study treatment. - Registered and compliant with the guidelines of the lenalidomide management programme (RevMate^®^). - Able to provide written informed consent. |
| **Exclusion Criteria** |
| - Women who are pregnant or breastfeeding. - The presence of another active malignancy or previous malignancy with a disease-free period. of <5 years, except for patients with carcinoma in situ or intramucosal carcinoma deemed to be cured by topical therapy. - Poorly controlled active thrombosis. - Refractory to either lenalidomide- and/or PI-based treatment regimen, defined as PD on therapy or PD ≤60 days after the last dose of therapy. Patients with PD 60 days after the last dose of therapy were considered relapsed. - Patients who have participated in a previous clinical trial of ixazomib or who have been previously treated with ixazomib. - Ongoing or active systemic infection, or known HBV, HCV, or HIV infection. - Major surgery*, radiation therapy, or infection ≤14 days† prior to enrollment in Treatment Period I (*surgery for bone lesions not considered major surgery; †if the radiation field was small, 7 days was considered sufficient). - Grade 1 peripheral neuropathy accompanied by pain, or Grade ≥2 peripheral neuropathy. - Current, uncontrolled cardiovascular conditions, including uncontrolled hypertension, uncontrolled cardiac arrhythmia, symptomatic congestive heart failure, unstable angina, or myocardial infarction within the past 6 months before enrolment. - CNS involvement. - Inability to swallow oral medications, inability or unwillingness to comply with the drug administration requirements, or gastrointestinal conditions that could interfere with the oral absorption or tolerance of treatment. - Psychiatric illness or other factors that would limit compliance with study treatment. - Comorbid systemic illnesses or other severe concurrent disease which, in the opinion of the investigator, would interfere with study participation or assessment of safety. |
| **IRd Treatment Period** |
| **Inclusion criteria** |
| - Received an injectable PI (bortezomib or carfilzomib) in each treatment cycle in the VRd or KRd treatment period. |
| **Exclusion criteria** |
| - Patients who do not achieve at least a minimal response to VRd or KRd in the VRd or KRd treatment period per IMWG response criteria. - Grade 1 peripheral neuropathy accompanied by pain, or Grade ≥2 peripheral neuropathy during the VRd or KRd treatment period. - Uncontrolled cardiovascular conditions, including uncontrolled hypertension, uncontrolled cardiac arrhythmia, symptomatic congestive heart failure, unstable angina, or myocardial infarction during the VRd or KRd treatment period. - Treatment with potent CYP3A4 inducing agents (i.e. rifampicin, rifapentine, rifabutin, carbamazepine, phenytoin, phenobarbital), or the use of gingko biloba or St. John’s wort. - Hypersensitivity to any of the IRd study medications, their analogues, or excipients contained in IRd. - Comorbid systemic illnesses or other severe concurrent disease which, in the opinion of the investigator, would interfere with study participation or assessment of safety. |

CNS, central nervous system; ECOG PS, Eastern Cooperative Oncology Group Performance Status; HBV, Hepatitis B virus; HCV, Hepatitis C virus; HIV, human immunodeficiency virus; IMWG, International Myeloma Working Group; IRd, ixazomib, lenalidomide, and dexamethasone; KRd, carfilzomib, lenalidomide, and dexamethasone; PD, progressive disease; PI, proteasome inhibitor; VRd, bortezomib, lenalidomide, and dexamethasone; RRMM, relapsing remitting multiple myeloma.

# Other secondary endpoints

*Methodology*

- - Relative dose intensity for each IRd study drug, defined as:
    - RDI = 100 (actual dose taken over the actual number of cycle days)

(total planned dose over the scheduled number of cycle days)

- - - The planned dose of IRd treatment per cycle was 4.0 mg x 3 for ixazomib, 25 mg x 21 for lenalidomide, and 40 mg x 4 for dexamethasone, in 28-day cycles.

*Results*

- The median (min, max) duration of IRd treatment was 22.2 (19.3, 25.5) months.
- Among the 11 patients who achieved CR, those with MRD <10^-4^ were 10 (91%) patients and 5 (56%) patients by the SRL-flow and NGS method, respectively.
- In addition, MRD was negative by the SRL-flow method (<10^-5^) in 7 (64%) patients and by the NGS method (<10^-6^) in 3 (33%) patients. MRD could not be measured by NGS in 2 patients.

## Supplementary Figure 1. Cumulative Best Response Over Time

#
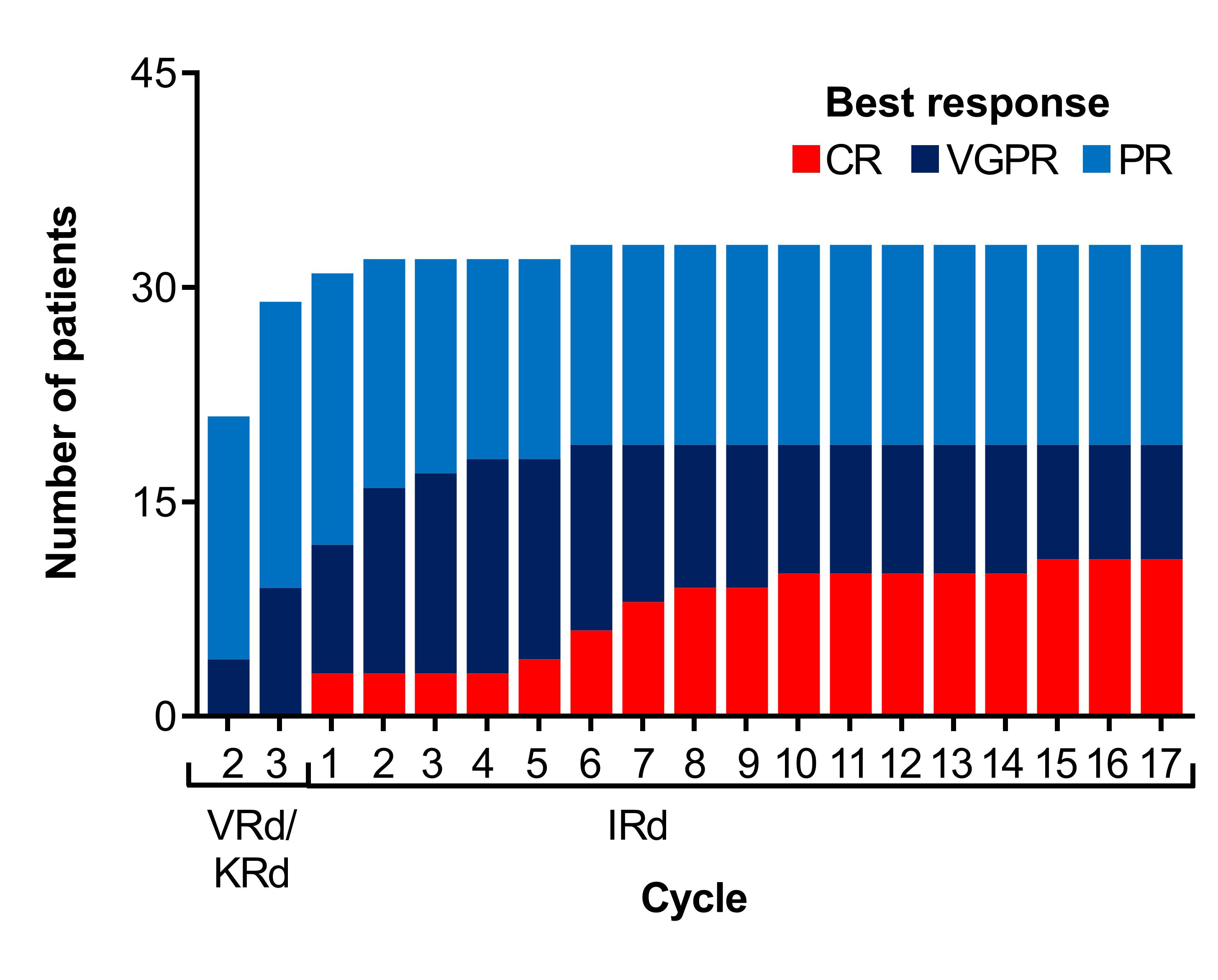


## Supplementary Figure 2. Relative dose intensity of ixazomib, lenalidomide, and dexamethasone during the study period (full analysis set)
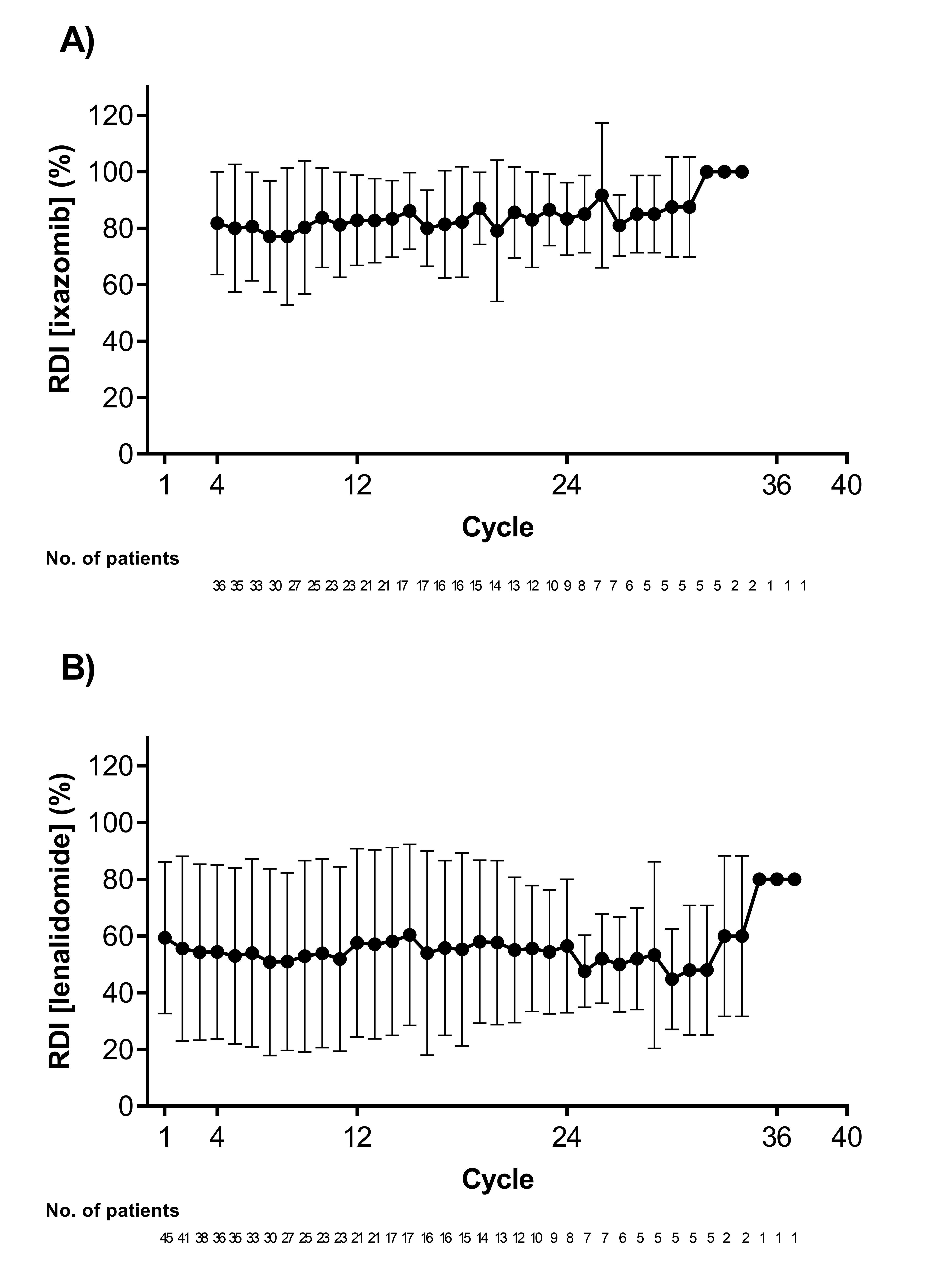


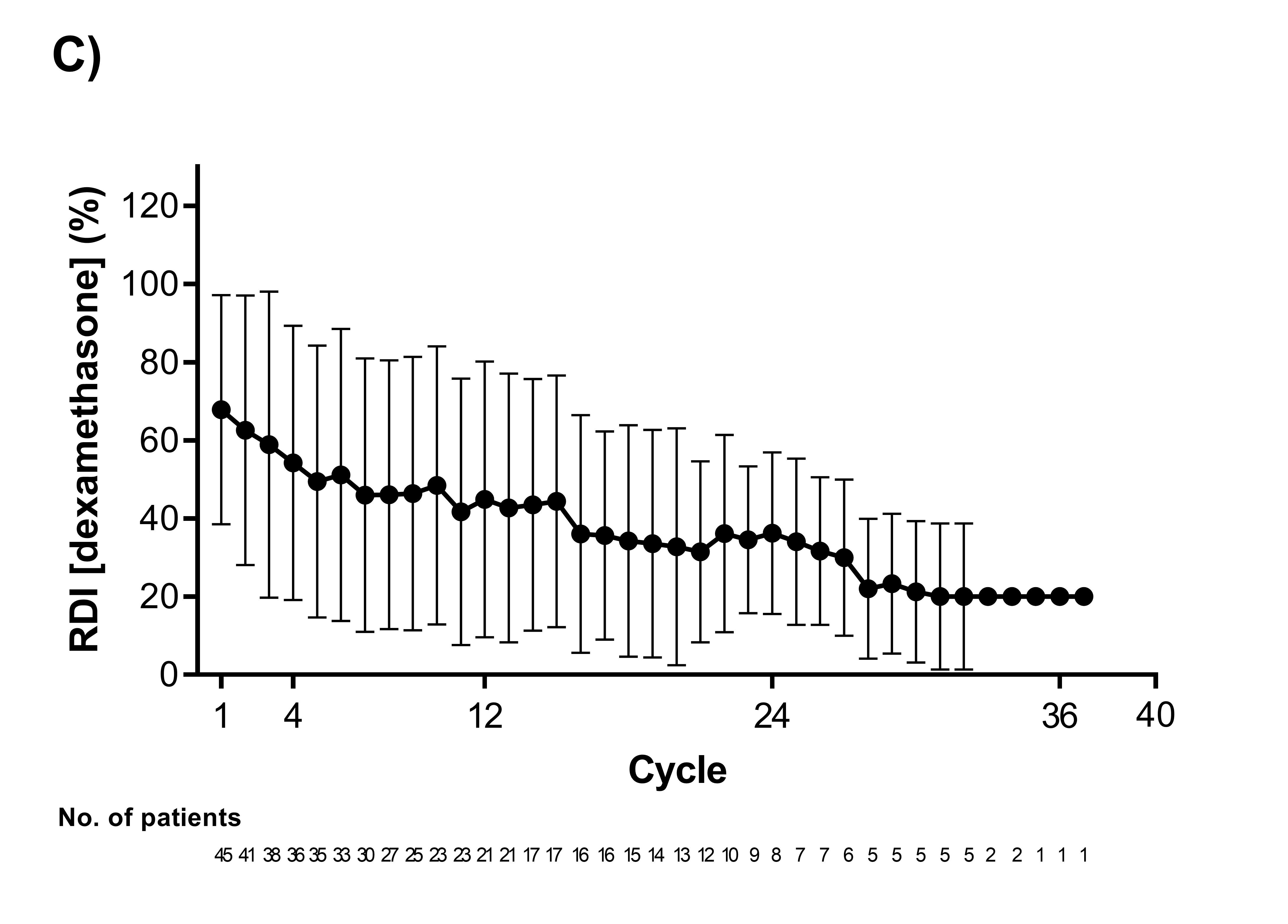


## Supplementary Figure 3. Time Plot of HRQOL Global Health Status (A), Disease Symptoms (B) Pain Symptom (C), Nausea and Vomiting (D), and Diarrhoea (E)


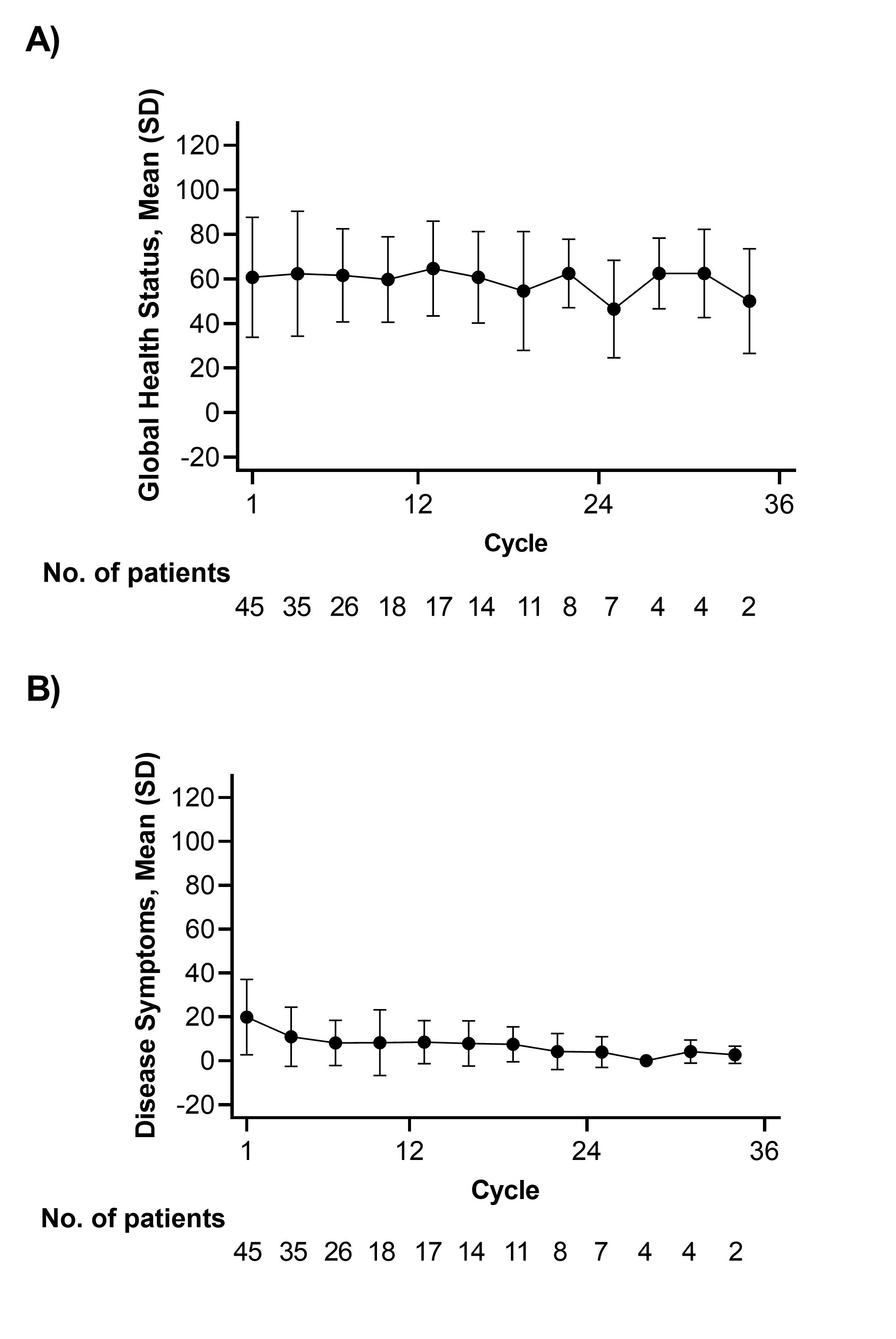


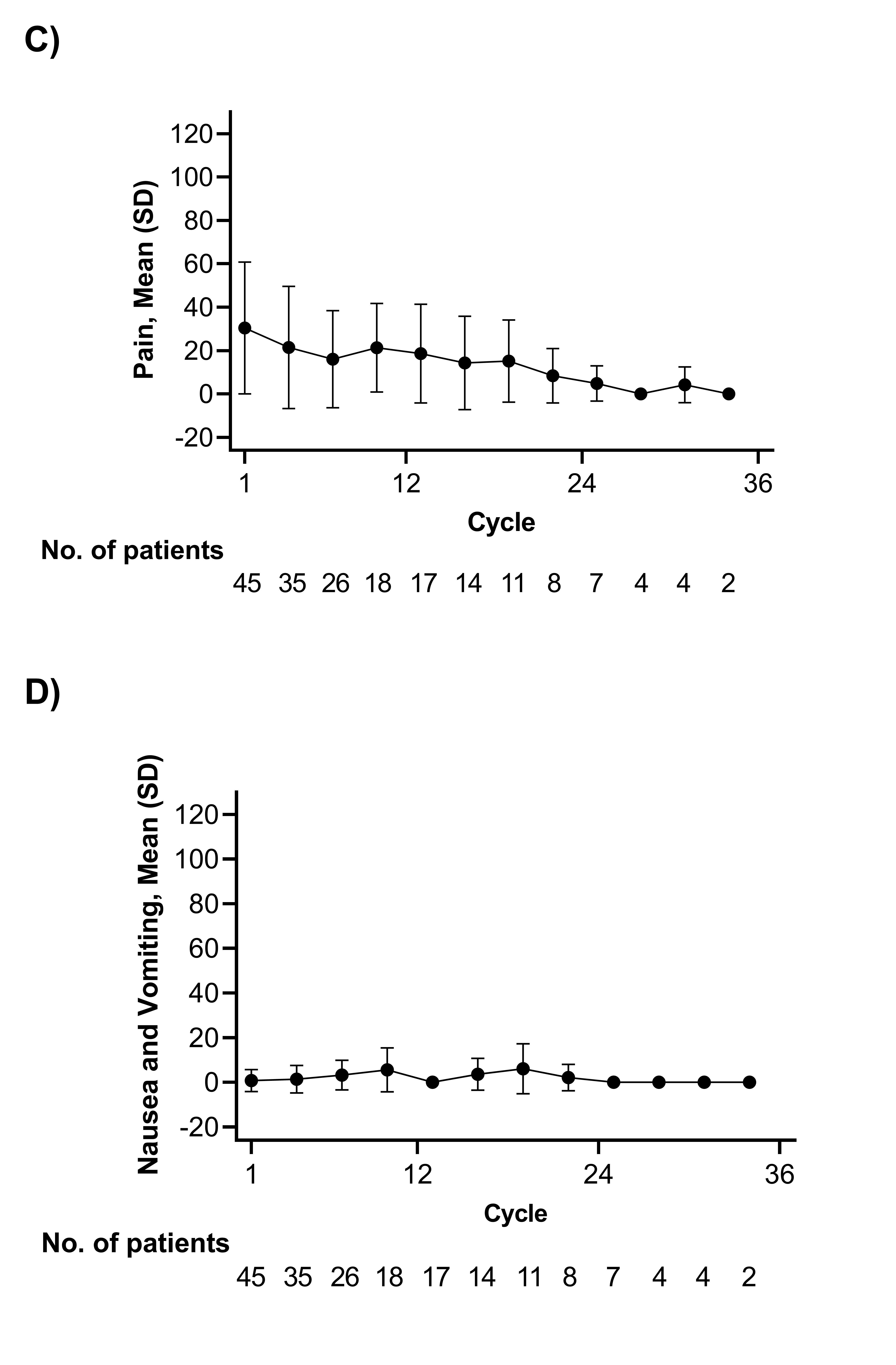


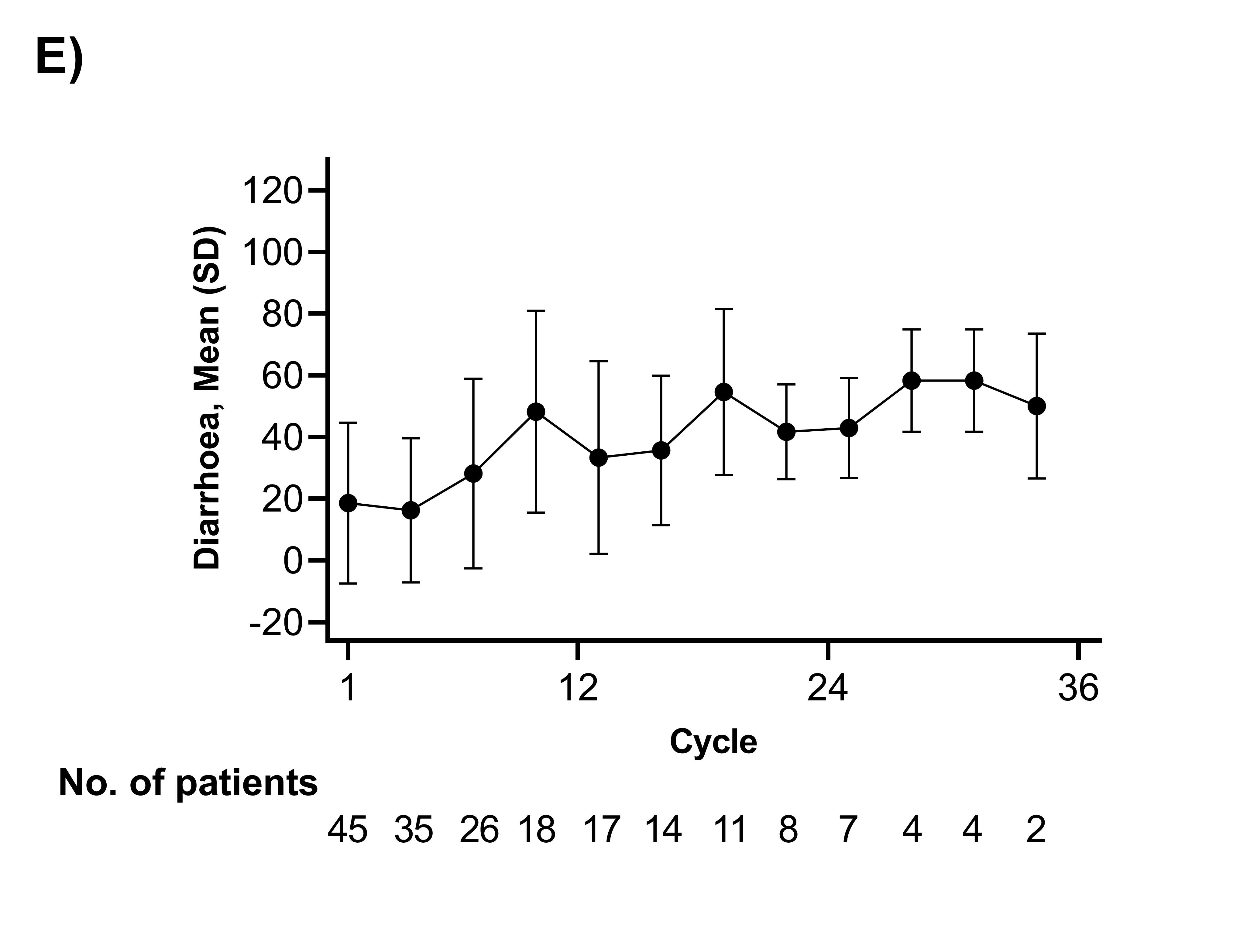

Supplement: Supplementary file 1 — (DOCX 2.93 mb) [file 277_2023_5212_MOESM1_ESM.docx]
