# Supplementary material for: Efficacy and Safety of Ixazomib Plus Lenalidomide and Dexamethasone Following Injectable PI-Based Therapy in Relapsed/Refractory Multiple Myeloma
Source: Ann Hematol. 2023 Jun 21;102(9):2493–504. doi: 10.1007/s00277-023-05212-7 (PMC10444638; doi:10.1007/s00277-023-05212-7)
Supplement: Supplementary file 2 — (DOCX 78.7 kb) [file 277_2023_5212_MOESM2_ESM.docx]

# Other secondary endpoints

*Methodology*

- - Relative dose intensity for each IRd study drug, defined as:
    - RDI = 100 (actual dose taken over the actual number of cycle days)

(total planned dose over the scheduled number of cycle days)

- - - The planned dose of IRd treatment per cycle was 4.0 mg x 3 for ixazomib, 25 mg x 21 for lenalidomide, and 40 mg x 4 for dexamethasone, in 28-day cycles.

*Results*

- The median (min, max) duration of IRd treatment was 22.2 (19.3, 25.5) months.
- Among the 11 patients who achieved CR, those with MRD <10^-4^ were 10 (91%) patients and 5 (56%) patients by the SRL-flow and NGS method, respectively.
- In addition, MRD was negative by the SRL-flow method (<10^-5^) in 7 (64%) patients and by the NGS method (<10^-6^) in 3 (33%) patients. MRD could not be measured by NGS in 2 patients.
