# Supplementary material for: Efficacy and Safety of Ixazomib Plus Lenalidomide and Dexamethasone Following Injectable PI-Based Therapy in Relapsed/Refractory Multiple Myeloma
Source: Ann Hematol. 2023 Jun 21;102(9):2493–504. doi: 10.1007/s00277-023-05212-7 (PMC10444638; doi:10.1007/s00277-023-05212-7)

## Supplementary Figure 2. Relative dose intensity of ixazomib, lenalidomide, and dexamethasone during the study period (full analysis set)
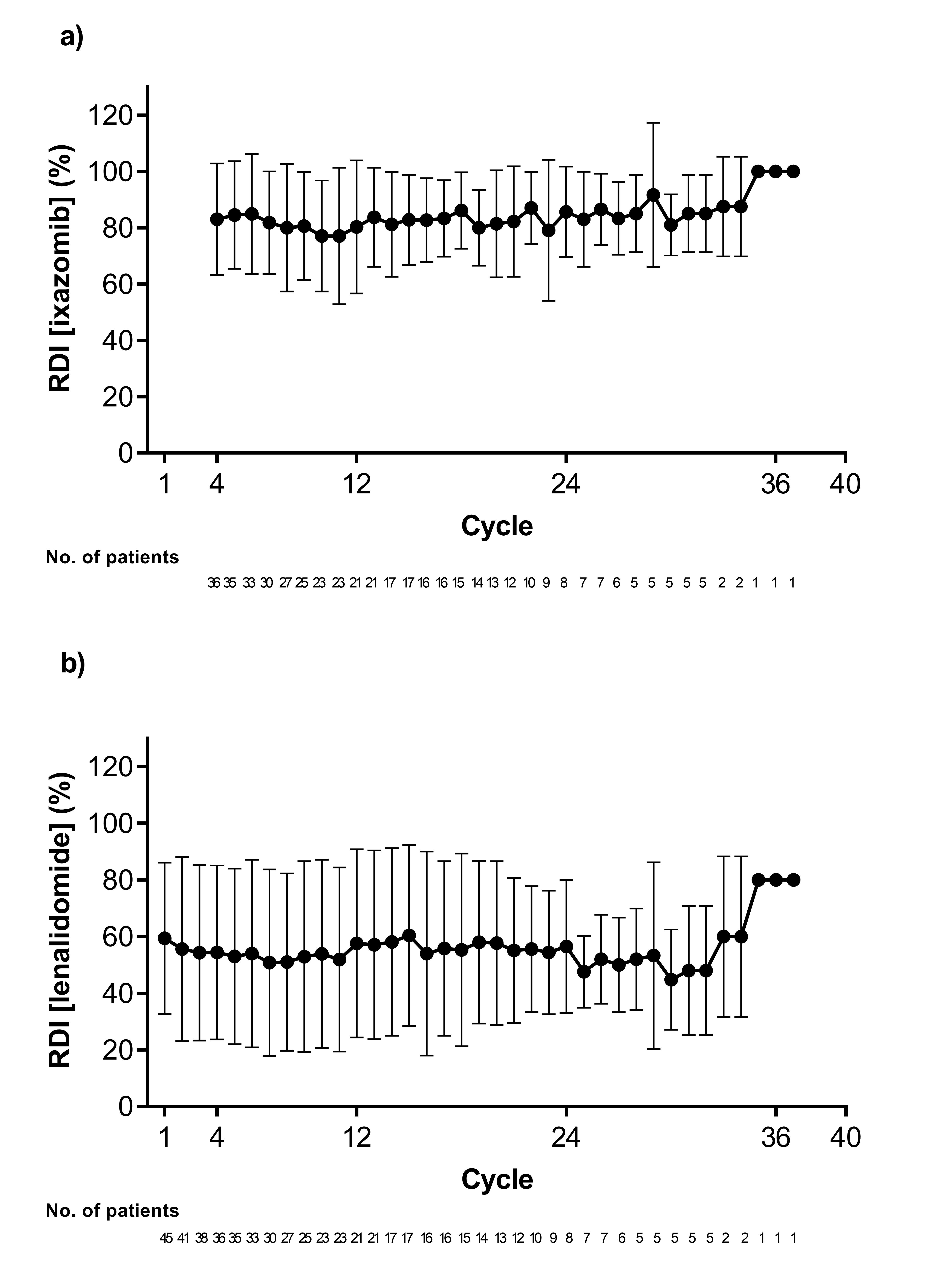


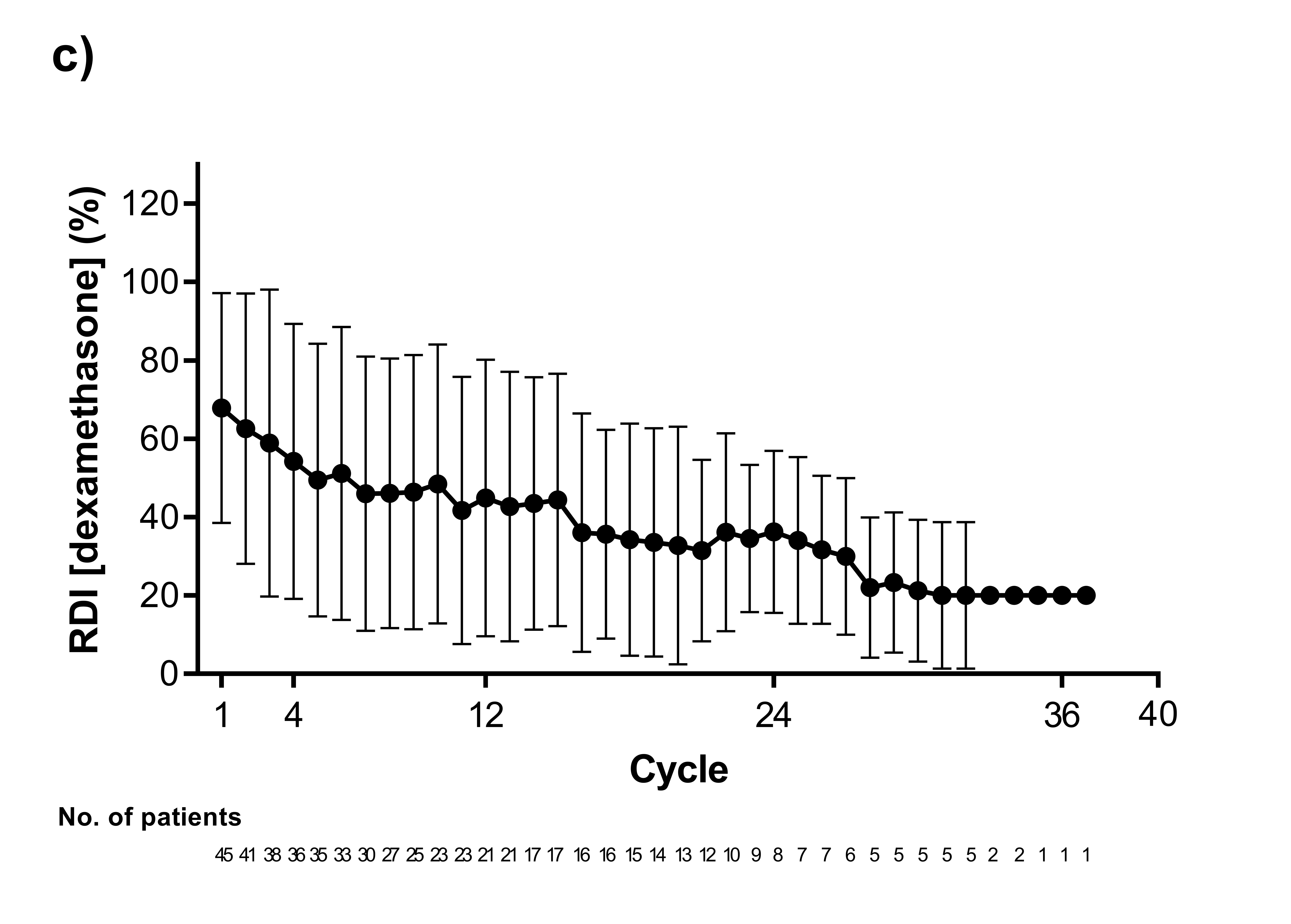

Supplement: Supplementary file 4 — (DOCX 3.19 mb) [file 277_2023_5212_MOESM4_ESM.docx]
