# Supplementary material for: Efficacy and Safety of Ixazomib Plus Lenalidomide and Dexamethasone Following Injectable PI-Based Therapy in Relapsed/Refractory Multiple Myeloma
Source: Ann Hematol. 2023 Jun 21;102(9):2493–504. doi: 10.1007/s00277-023-05212-7 (PMC10444638; doi:10.1007/s00277-023-05212-7)

## Supplementary Figure 3. Time Plot of HRQOL Global Health Status (A), Disease Symptoms (B) Pain Symptom (C), Nausea and Vomiting (D), and Diarrhoea (E)








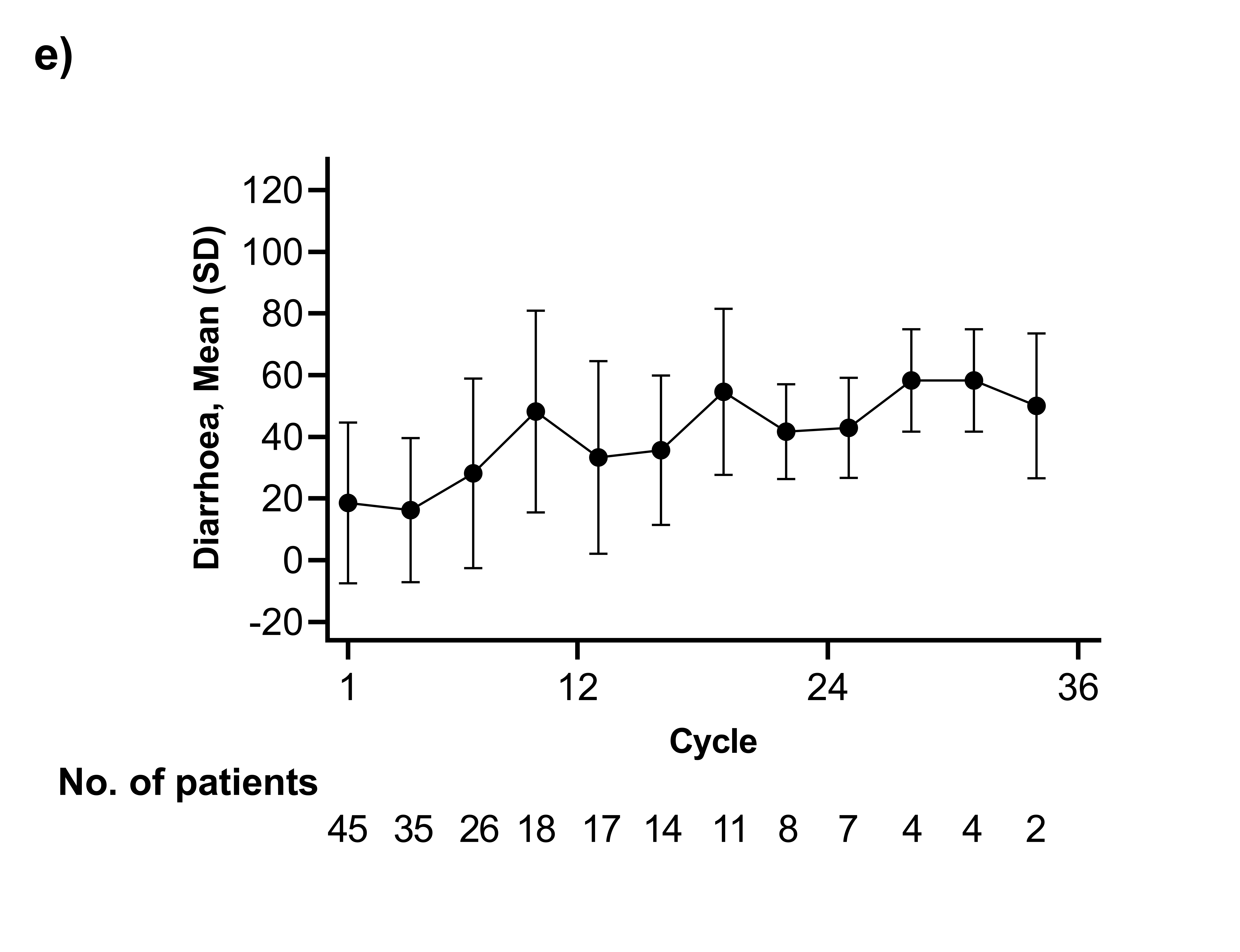

Supplement: Supplementary file 5 — (DOCX 9.76 mb) [file 277_2023_5212_MOESM5_ESM.docx]
